# Regional variations and predictors of low uptake of cervical cancer screening among women aged 30–49 years in Tanzania: a geospatial and multilevel analysis of population-based survey

**DOI:** 10.1007/s43999-026-00095-3

**Published:** 2026-08-06

**Authors:** Deogratius Bintabara, Costantine C. Kamata, Ramadhani Mohamedi, Eveline Konje, Athanase Lilungulu

**Affiliations:** 1 Department of Community Medicine, School of Medicine and Dentistry, The University of Dodoma, Dodoma, Tanzania; 2 Department of Anatomy and Histology, School of Medicine and Dentistry, The University of Dodoma, Dodoma, Tanzania; 3 Department of Information Systems and Technology, College of Informatics and Virtual Education, The University of Dodoma, Dodoma, Tanzania; 4 Department of Epidemiology and Biostatistics, School of Public Health, Catholic University of Health and Allied Sciences, Mwanza, Tanzania; 5 Department of Obstetrics and Gynecology, School of Medicine and Dentistry, The University of Dodoma, Dodoma, Tanzania

**Keywords:** Cervical cancer, Screening, Low uptake, Regional variations, Tanzania

## Abstract

**Introduction:**

More than 90% of the highest incidence rates of cervical cancer occur in sub-Saharan Africa region. However, this region has low uptake of cervical cancer screening compared to other World Health Organization (WHO) regions. Therefore, the current study aimed to show the geographical and regional variation and predictors of low uptake of cervical cancer screening among women aged 30–49 years in Tanzania.

**Methods:**

This study analyzed data from 2022 Tanzania Demographic and Health Survey and Malaria Indicator Survey (TDHS-MIS). The uptake of cervical cancer screening was the outcome variable which dichotomized into “Yes” for the women who reported a doctor or other healthcare provider ever tested her for cervical cancer otherwise it was coded “No.” Besides, descriptive and geospatial analysis, the mixed-effect multilevel logistic regression analysis was performed to determine predictors of low uptake of cervical cancer screening.

**Results:**

This study included a total of 6911 women between 30 and 49 years old, with the median age (interquartile range) of 38 (34–43) years. The overall uptake of cervical cancer in Tanzania remain low (12.2%). The geospatial analysis indicates the lowest uptake was observed in Southern (4.7%) and Central (5.9%). The random effect model revealed that the variation in uptake of cervical cancer screening, 26% was attributed to between clusters within regions or geographical zones differences [ICC = 0.26, 95%CI; 0.22–0.31]. Fixed effect model revealed that women with older ages, formal education, health insurance coverage, employed for cash, being in rich household, listen to radio, reading newspapers, magazines, or using the internet had higher odds of uptake cervical cancer screening than their counterparts.

**Conclusion:**

For realization of WHO target on elimination of cervical cancer by screening 70% of all eligible women age 30 and above by 2030, more efforts on addressing social determinants of health and provision of health education through mass media should be considered.

**Clinical trial number:**

Not applicable.

## Introduction

Cervical cancer remains one of the leading causes of cancer-related morbidity and mortality among women worldwide, with 662,301 new cases and 348,874 deaths reported in 2022 [1]. This burden is disproportionately higher in low- and middle-income countries (LMICs), particularly in Africa and Asia, where incidence and mortality rates are among the highest globally [1]. In Africa, Tanzania has one of the highest incidence and mortality rates, with approximately 11,000 new cases and 7,000 deaths reported in 2022, maintaining its position as the leading cause of cancer-related morbidity and mortality among Tanzanian women [1].

In response to this growing public health challenge, the World Health Organization (WHO) introduced a global strategy in 2020, aimed at accelerating the elimination of cervical cancer. The strategy established the ambitious 90-70-90 targets to be achieved by 2030. These targets are: (i) 90% of girls fully vaccinated against Human Papilloma Virus (HPV) by the age of 15, (ii) 70% of women screened with a high-performance test by the age 35 and a repeat by the age 45, and (iii) 90% of women diagnosed with cervical disease—including precancerous lesions and invasive cancers—receiving appropriate treatment [2].

As part of its national cancer prevention and control efforts, Tanzania has made significant strides in line with the WHO’s strategy. In collaboration with key stakeholders, the country integrated the HPV vaccine into the national immunization program for 14-year-old girls in 2018, following a successful pilot trial in the Kilimanjaro region that began in 2014 [3, 4]. To date, vaccine coverage stands at 79% for the first dose and 60% for the second dose [5, 6]. In addition to vaccination, Tanzania has prioritized cervical cancer screening services, with the cost-effective method, visual inspection with acetic acid (VIA) widely available at primary and higher-level healthcare facilities and through government-led and stakeholder campaigns [7–9]. In more resource-equipped settings, additional highly efficacious screening methods like the HPV testing and Papanicolaou smear cytology are also employed [10, 11].

Despite these efforts, there remains a significant gap in screening coverage, with varying uptake rates ranging from 6% to 21% in diverse study populations [12, 13]. These gaps likely contribute to the persistently high proportion of cervical cancer cases diagnosed at advanced stages, leading to poor treatment outcomes [14, 15].

Various isolated and localized studies have highlighted potential barriers to screening uptake, including limited awareness and knowledge on cervical cancer and the role of screening, fear, stigma, cultural beliefs, healthcare access challenges, and socioeconomic factors [16–19]. However, these studies while valuable, lack of comprehensive, broad perspective on national and regional cervical cancer screening coverage, and its individual and community-level determinants.

This paper addresses this gap by presenting findings from a secondary analysis of nationally representative data from the 2022 Tanzania Demographic and Health Survey and Malaria Indicator Survey (TDHS-MIS) among women aged 30–49. The analysis highlights geographic disparities in cervical cancer screening uptake and identifies key predictors at both the individual and community levels. These findings offer crucial evidence for informing more targeted public health interventions, such as equitable resource allocations and capacity building initiatives, to improve screening coverage and reduce the burden of cervical cancer across diverse regions in Tanzania.

## Methods

### Data source

This study analysed the data from the 2022 Tanzania Demographic and Health Survey and Malaria Indicator Survey (2022 TDHS-MIS). This 2022 TDHS-MIS was conducted by the National Bureau of Statistics (NBS) and the Office of the Chief Government Statistician Zanzibar (OCGS) in collaboration with the Ministries of Health (MoH) in Tanzania Mainland and Zanzibar. The Tanzania Food and Nutrition Centre (TFNC) collaborated on several aspects of the survey, especially biomarkers. Data collection took place from February to July 2022. The technical support for the survey was provided by ICF International under the Demographic and Health Survey (DHS) program. These surveys have been conducted after every four years since 1990. The survey collected information from a nationally-representative sample of women aged 15–49 years in the selected households.

### Study design

The current study analyzed a recent dataset from nationwide population-based cross-sectional survey using information obtained by interviewing women of reproductive age who were either residents or visitors in the household on the night before the survey.

### Sample size and sampling procedure

**Fig. 1:**
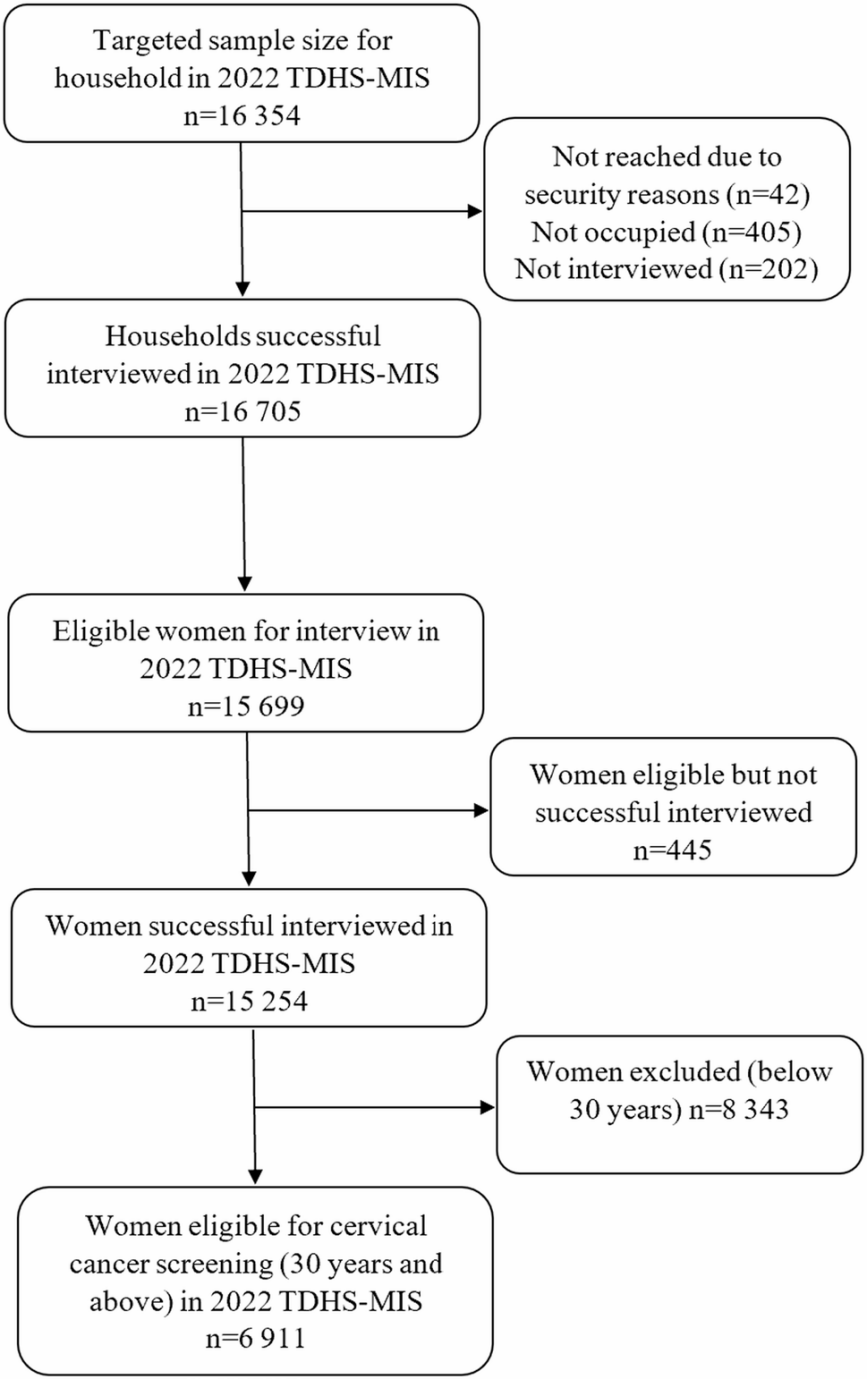
Selection of study respondents

The 2022 TDHS-MIS employed a stratified two-stage cluster sampling techniques to obtain a sample intended to provide estimates for the entire country, for urban and rural areas in Tanzania Mainland, and for Zanzibar. The first stage involved selection of sampling points (clusters) consisting of enumeration areas (EAs) delineated for the 2012 Tanzania Population and Housing Census [20]. A total of 629 clusters were selected by using probability proportional to size within each sampling stratum. In the second stage, 26 households were selected systematically from each cluster, for a total targeted sample size of 16,354 households for the 2022 TDHS-MIS. However, one EA were not reached due to security reasons, therefore a total of 16,312 households were selected in which 15,907 were found to be occupied, but only 15,705 were successfully interviewed. In the interviewed households, 15,699 women age 15–49 were identified as eligible for individual interviews but interviews were successful completed with 15,254 women, yielding a response rate of 97%. After excluding women who were less than 30 years (not eligible for mandatory cervical cancer screening in Tanzania), a final sample size of 6911 women aged 30–49 was included in this analysis (Fig. 1).

### Data collection and processing

The TDHS-MIS used five questionnaires during data collection. However, this study used data collected using the Women’s Questionnaire. After the pretesting of the questionnaires, the finalized and corrected version was used in the main surveys. Data collection was performed by trained and qualified interviewers through a series of practical tests and examinations. Data were collected by using computer-assisted personal interviewing (CAPI) from 24 February to 21 July 2022 (5 months). The data processing of the 2022 TDHS-MIS ran concurrently with the data collection exercise. The electronic data files from each completed cluster were transferred via Syncloud to the NBS central office server in Dodoma. The data files were registered and checked for inconsistencies, incompleteness, and outliers. Errors and inconsistencies were communicated to the field teams for review and correction. Finally, data editing and cleaning were performed.

### Measurement of variable

#### Dependent variable

The uptake of cervical cancer screening was the dependent variable in this study. This variable was coded ‘Yes’ for women who reported having ever undergone cervical cancer screening by a healthcare professional and ‘No’ otherwise. During cervical cancer screening, the woman is positioned in the lithotomy position to allow visualization of the cervix. Then, either of the following two screening tests is performed; (i) the health care provider uses a brush or swab to collect a sample from inside her and the sample is sent to a laboratory for testing (this test is called a Pap smear or human papillomavirus (HPV) test) or (ii) healthcare provider uses a visual inspection with acetic acid (VIA). In this test, the health care provider puts vinegar on the cervix to see if there is a reaction.

#### Explanatory variables

Following a detailed literature review on factors associated with uptake of preventive services for women of reproductive age [21–23], a total of thirteen explanatory variables available in 2022 TDHS-MIS were included in this analysis. These variables were grouped into individuals or households’ variables (level 1) and clusters variables (level 2). The level 1 variables were age (30–34, 35–39, 40–49), marital status (never married, married/living together, separated/widow/divorced), education level (none, primary, secondary, tertiary), employed in last 12 months (not employed, employed for cash, employed but paid-in-kind), covered by health insurance (yes, no), parity (nullipara, primipara, multipara), reading newspaper/magazine (yes, no), listening to radio (yes, no), watching television (yes, no), use of internet in last 12 months (yes, no) and number of problems in accessing healthcare as a composite variable, with five categories (none, one, two, three and four) created based on the number of the following problems reported in accessing healthcare: (i) obtaining permission to go to the doctor, (ii) obtaining money to pay for advice or treatment, (iii) distance to a health facility and (iv) not wanting to go alone. The level 2 variables were household wealth status (poor, middle, rich), geographical zone (central, coastal, lake, northern, southern, southern highlands, western, Zanzibar) and residence (rural, urban).

### Statistical analysis

Descriptive analysis, geospatial analysis and mixed-effect multilevel logistic regression analysis have been performed in this study. In descriptive analyses, all categorical variables were summarized using frequencies and percentages and then presented in either tables or graphs while continuous variable were summarized by using median and interquartile range (IQR). The geospatial analysis was used to create spatial map which show the proportion of women with uptake of cervical cancer screening by zones and regions. Finally, a mixed-effects multilevel logistic regression analysis was performed to account for the hierarchical structure of the data whereby individuals or households (level 1) were nested within clusters (level 2). These multilevel models were fitted to identify factors associated with uptake of cervical cancer screening. A total of four models with the dependent variable “uptake of cervical cancer screening” were estimated. In the Model I (empty model) no explanatory variable was added. This model showed the changes in uptake of cervical cancer screening attributed to variations between clusters. In Model II, the individual level variables were included, while in Model III, the cluster level variables were included. In Model IV, both individual variables and cluster variables were included. The mixed-effect regression analysis provided results that included both fixed effects and random effects. The fixed effect results from Model II, III, and IV were presented as odds ratios (OR) with their corresponding confidence interval (CI), while the results of the random effects were presented as the variance which indicates variations in uptake of cervical cancer screening. The variance random component of the models was estimated by calculating the variance of the cluster-level variations and their corresponding standard errors. The Intra-class correlation (ICC) was calculated to evaluate whether the variations in the uptake of cervical cancer are primarily within or between the clusters. Since the individual (level 1) were nested within the clusters (level 2), the Chi-square likelihood–ratio test was used to assess the difference between models. The p-values were estimated using Wald statistics and *p* < 0.05 was taken to indicate statistical significance. The statistical analyses were performed using Stata 17 (Stata Corp, College Station, TX). The “svy” set command was used to adjust for the complex sampling design used by TDHS-MIS. All estimates were weighted to correct for non-responses and disproportionate sampling. The generalized variance inflation factor (VIF) was performed to test for multi-collinearity, which usually should not exceed 5. In this analysis no variable presented with VIF > 3.0, suggesting no suspicions for multi-collinearity.

## Results

### Baseline characteristics of the respondents

Table 1 presents the baseline characteristics of the respondents in which a total of 6911 women between 30 and 49 years old were interviewed and included in the analysis. The median age (IQR) of the respondents was 38 (34–43) years. Majority (74.94%) of the respondents were living with their spouse at the time of the interview. More than 20% did not attended any formal education. Nearly quarter (22.47%) were not employed in the last 12 months before the interview and less than 8% reported having any type of health insurance. Majority (90.15%) of the respondents were multiparas. Few respondents were exposed to media sources in which about 30% of respondents were reported to listen radio and/or watching television while less than 15% reported to use internet in the last 12 months before the interview. About one-third of the respondents were from rural area and communities in households with poor wealth status while more than half reported having at least one problem in accessing healthcare.

**Table 1:** Percent distribution of women aged 30 to 49 years by selected background characteristics, TDHS-MIS (*n* = 6911)

Variable	*n* (%) weighted
**Age** (median (IQR) = 38 (34–43))	
30–34	2076 (30.04)
35–39	1884 (27.26)
40–49	2951 (42.69)
**Marital status**	
Never married	360 (5.20)
Married/living together	5179 (74.94)
Separated/widow/divorced	1372 (19.86)
**Education level**	
None	1504 (21.76)
Primary	4289 (62.07)
Secondary	1011 (14.62)
Tertiary	109 (1.55)
**Employed in last 12 months**	
Not employed	1553 (22.47)
Employed for cash	3577 (51.76)
Employed but paid-in-kind	1781 (25.77)
**Covered by health insurance**	
No	6409 (92.73)
Yes	502 (7.27)
**Parity** (median (IQR) = 4 (3–6))	
Nullipara	254 (3.67)
Primipara	427 (6.18)
Multipara	6230 (90.15)
**Reading newspaper/magazine**	
No	6472 (93.65)
Yes	439 (6.35)
**Listening to radio**	
No	4755 (68.80)
Yes	2156 (31.20)
**Watching television**	
No	5014 (72.56)
Yes	1897 (27.44)
**Use of internet in last 12 months**	
No	5993 (86.71)
Yes	918 (13.29)
**Household wealth status**	
Poor	2319 (33.56)
Middle	1352 (19.57)
Rich	3240 (46.87)
**Number of problems in accessing healthcare**	
None	3345 (48.40)
One	1779 (25.74)
Two	1045 (15.12)
Three	565 (8.18)
Four	177 (2.57)
**Geographical zone**	
Central	522 (7.56)
Coastal	1574 (22.77)
Lake	1738 (25.15)
Northern	651 (9.42)
Southern	597 (8.64)
Southern highlands	794 (11.49)
Western	735 (10.63)
Zanzibar	300 (4.34)
**Residence**	
Rural	2420 (35.02)
Urban	4491 (64.98)

### Uptake of cervical cancer screening

**Fig. 2:**
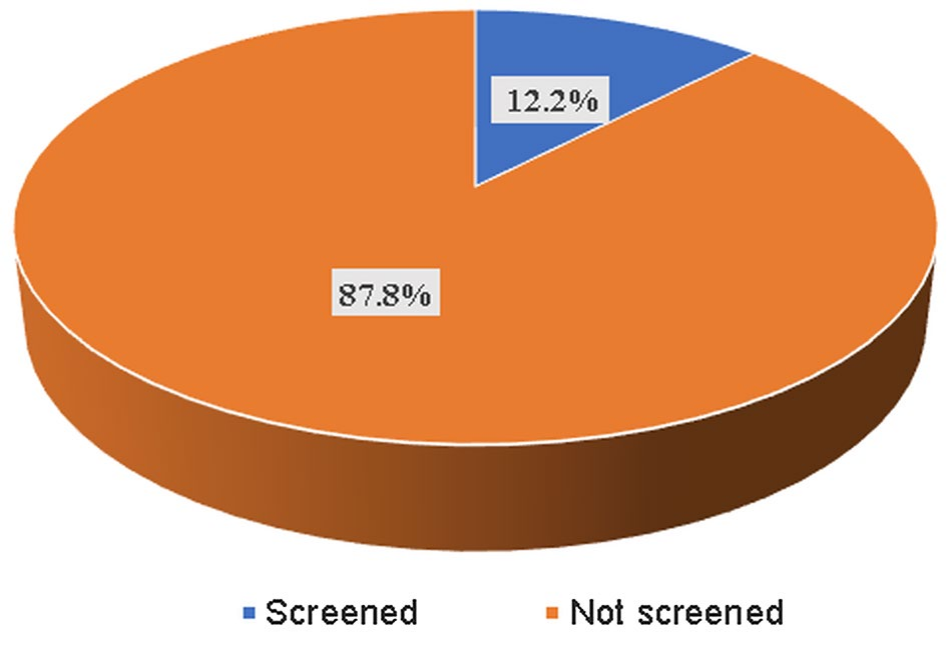
Percentage distribution of uptake of cervical cancer screening among women aged 30–49 years, TDHS-MIS 2022 (*n* = 6911)

Figure 2 shows the percentage distribution of uptake of cervical cancer screening among women aged 30–49 in Tanzania. The findings revealed that only 12% of the respondents reported to uptake cervical cancer screening.

### Uptake of cervical cancer screening by geographical zones and regions

**Fig. 3:**
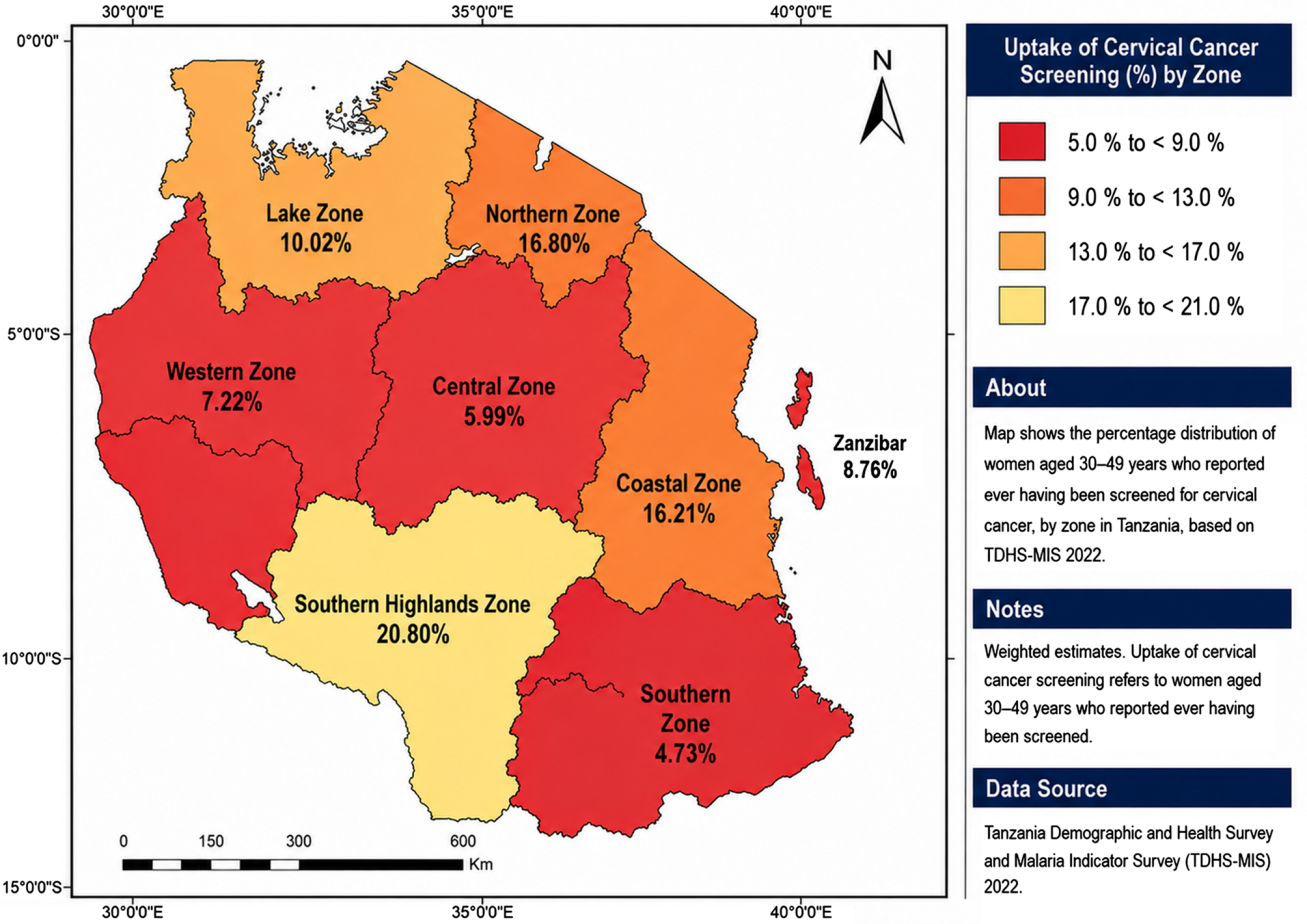
Percentage distribution of uptake of cervical cancer screening by zones among aged 30–49 years, TDHS-MIS 2022 (*n* = 6911)

**Fig. 4:**
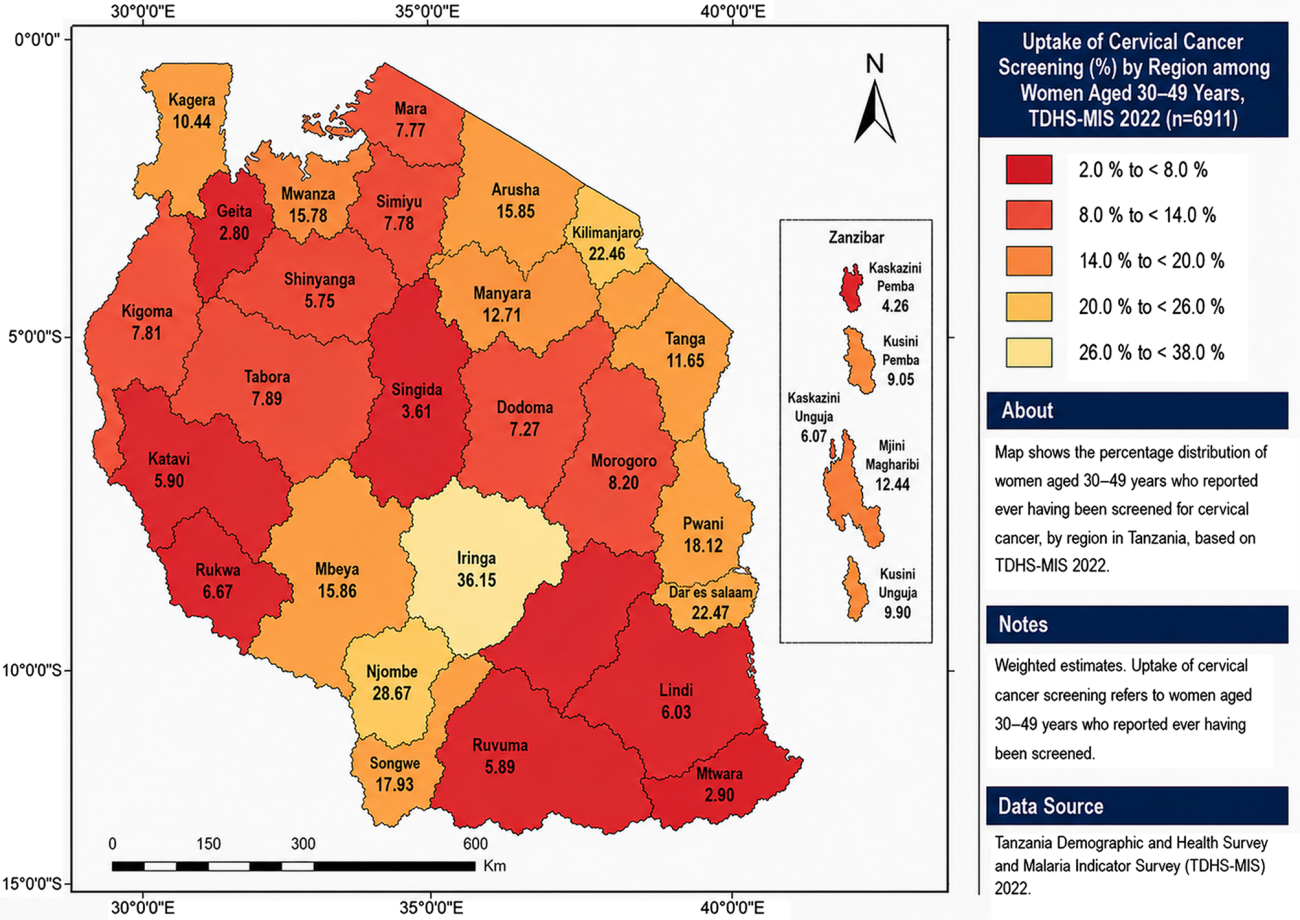
Percentage distribution of uptake of cervical cancer screening by regions among aged 30–49 years, TDHS-MIS 2022 (*n* = 6911)

Figure 3 presents the output of geospatial analysis indicating the proportion of uptake of cervical cancer screening by zones among women aged 30–49 years in Tanzania. The lowest uptake was observed in in Southern (4.73%), Central (5.99%), Western (7.22%) and Zanzibar (8.76%) zones of Tanzania. After disintegrated the results into regions, it was observed that the regions with lowest uptake of cervical cancer screening were Geita (2.80%), Mtwara (2.90%), Singida (3.61%) and Kaskazini Pemba (4.26%) as shown in Fig. 4.

### Predictors of low uptake of cervical cancer screening

Table 2 presents the results of a mixed-effects multilevel logistic regression of the association between outcome variable (uptake of cervical cancer screening) and several identified predictors as explanatory variables.

#### Fixed effect part results

The final model IV which include the individual level and cluster level variables show the predictors of uptake of cervical cancer screening among women aged 30 to 49 years in Tanzania. The findings indicate that, the odds of uptake cervical cancer screening were nearly two times higher among older women aged 40 to 49 years [AOR = 1.69, 95%CI; 1.28–2.22] compared to younger women aged 30 to 34 years and among separated/widow/divorced women [AOR = 1.64, 95%CI; 1.03–2.61] compared to those who were never married. Similarly, the odds of uptake cervical cancer screening were nearly two times higher among women with primary [AOR = 1.60, 95%CI; 1.15–2.24] and secondary education [AOR = 1.95, 95%CI; 1.28–2.96] compared with those without any formal education. Furthermore, the odds of uptake cervical cancer screening were nearly 1.5 times higher among women reported having employed for cash [AOR = 1.39, 95%CI; 1.05–1.84] and those who covered with any type of health insurance [AOR = 1.39, 95%CI; 1.01–1.94] compared with those not employed and without health insurance respectively. Additionally, women who were reported to listen radio [AOR = 1.37, 95%CI; 1.10–1.69] and those reported to use internet in the last 12 months [AOR = 1.62, 95%CI;1.22–2.17] had higher odds of uptake cervical cancer screening compared with their counterparts. Moreover, women from rich households [AOR = 2.28, 95%CI; 1.63–3.20] and urban residence [1.64, 95%CI; 1.27–2.17] had higher odds of uptake cervical cancer screening compared those from poor and rural residence respectively.

#### Random effect part results

The random effects model I (empty model) showed significant variability in the odds of uptake cervical cancer screening between clusters [σ^2^ = 0.09, 95%CI; 0.08–0.11]. Furthermore, the model revealed that more than 25% of uptake of cervical cancer screening was attributed to the variations between the clusters [ICC = 0.26, 95%CI; 0.22–0.31].

**Table 2:** Multilevel logit models for the factors associated with breast cancer screening among women aged 30–49 year, TDHS-MIS 2022 (*n* = 6911)

Variable	Model 1 AOR [95% CI]	Model 2 AOR [95% CI]	Model 3 AOR [95% CI]	Model 4 AOR [95% CI]
**Fixed Effects Component**				
**Level 1 variables**				
**Age** (ref: 30–34)				
35–39		1.27 [0.95–1.71]		1.26 [0.94–1.68]
40–49		**1.70 [1.28–2.24]**		**1.69 [1.28–2.22]**
**Marital status** (ref: Never married)				
Married/living together		1.35 [0.88–2.06]		1.44 [0.94–2.21]
Separated/widow/divorced		**1.58 [1.01–2.49]**		**1.64 [1.03–2.61]**
**Education level** (ref: None)				
Primary		**1.97 [1.43–2.71]**		**1.60 [1.15–2.24]**
Secondary		**2.52 [1.68–3.78]**		**1.95 [1.28–2.96]**
Tertiary		**2.43 [1.21–4.89]**		1.90 [0.95–3.80]
**Employed in last 12 months** (ref: Not employed)				
Employed for cash		**1.47 [1.11–1.94]**		**1.39 [1.05–1.84]**
Employed but paid-in-kind		0.95 [0.68–1.31]		1.01 [0.73–1.40]
**Covered by health insurance** (ref: No)				
Yes		**1.41 [1.01–1.98]**		**1.39 [1.01–1.94]**
**Parity** (ref: Nullipara)				
Primipara		1.00 [0.59–1.68]		0.97 [0.57–1.66]
Multipara		0.73 [0.47–1.12]		0.72 [0.46–1.13]
**Reading newspaper/magazine** (ref: No)				
Yes		1.00 [0.68–1.47]		0.95 [0.65–1.39]
**Listening to radio** (ref: No)				
Yes		**1.52 [1.23–1.88]**		**1.37 [1.10–1.69]**
**Watching television** (ref: No)				
Yes		1.14 [0.90–1.45]		0.91 [0.71–1.15]
**Use of internet in last 12 months** (ref: No)				
Yes		**1.87 [1.39–2.54]**		**1.62 [1.22–2.17]**
**Number of problems in accessing healthcare** (ref. None)				
One		0.99 [0.78–1.25]		1.09 [0.86–1.38]
Two		1.14 [0.78–1.66]		1.36 [0.94–1.97]
Three		0.91 [0.60–1.38]		1.20 [0.79–1.80]
Four		**0.30 [0.10–0.93]**		0.35 [0.11–1.08]
**Level 2 variables**				
**Household wealth status** (ref: poor)				
Middle			1.35 [0.97–1.89]	1.23 [0.87–1.73]
Rich			**3.14 [2.30–4.29]**	**2.28 [1.63–3.20]**
**Geographical zone** (ref: Central)				
Coastal			**2.06 [1.33–3.20]**	**2.24 [1.45–3.47]**
Lake			**1.65 [1.06–2.57]**	**1.79 [1.17–2.76]**
Northern			**3.34 [2.11–5.30]**	**3.42 [2.18–5.36]**
Southern			0.94 [0.52–1.69]	1.07 [0.59–1.94]
Southern highlands			**4.72 [3.01–7.41]**	**4.63 [3.01–7.14]**
Western			1.44 [0.85–2.44]	**1.82 [1.09–3.06]**
Zanzibar			1.35 [0.86–2.09]	1.29 [0.81–2.05]
**Residence** (ref: Rural)				
Urban			**1.78 [1.38–2.29]**	**1.64 [1.27–2.17]**
*Random Effects Component*				
Level 2 (community) variance	0.09 [0.08–0.11]	0.65 [0.46–0.91]	0.45 [0.30–0.67]	0.40 [0.26–0.62]
Intraclass correlation	0.26 [0.22–0.31]	0.16 [0.12–0.22]	0.12 [0.08–0.17]	0.11 [0.07–0.16]
Akaike’s information criterion	4857.41	4637.28	4606.09	4498.05
Log pseudolikelihood	-2426.71	-2296.64	-2291.04	-2217.03

## Discussion

The study findings suggest a low uptake of cervical cancer screening in Tanzania with approximately only twelve of a hundred (12.6%) women in reproductive age group reported utilizing available screening services. Although, most of low and middle income countries [24] have reported low uptake of cervical cancer screening for instance Eastern Africa countries like Kenya (16.4%) [25] and Uganda (20.6%) [26], the prevalence in Tanzania is still lower (12.6%). However, the estimated uptake of cervical cancer screening in this study is similar to the pooled prevalence (12.87%) in sub-Saharan Africa as reported in meta-analysis by Yimer et al. [24]. The reasons for low uptake of cervical cancer screening are related to lack of knowledge on seriousness of the disease, general knowledge about cervical cancer, and mostly sociocultural and health system related factors [26–30]. In addition, integrating HPV vaccination programs for children with cervical cancer screening opportunities for eligible mothers may provide a synergistic approach to improving coverage, particularly by leveraging existing school- and community-based health platforms to reach both adolescents and adult women. This situation may have public health implication in fighting to reduce burden of cervical cancer among women in reproductive age group. For elimination of cervical cancer among reproductive women globally, WHO through a global health strategy initiative call upon countries to eliminate cervical cancer with proposed target of 70% of women to be screened in their 35 years and re-screened in their 45 years [31].

For Tanzania to achieve this target, not only services should be accessible, available, and affordable but also culturally acceptable and sound. It has been reported that women consider cancer screening services as culturally unacceptable because of the procedure itself and most of health workers are men which jeopardize the privacy of women [24, 30]. Tanzania remains to be among the LMICs with high incidence and mortality attributed to cervical cancer [32] which is explained by late presentation of most of cervical cancer patients that affects the treatment plan and prognosis [33]. On the other hand, the health facility readiness on provision of cervical cancer screening remains low at 36% as reported in SARA 2023 report [34].

Furthermore, in this study, considerable difference was observed on uptake of cervical cancer screening across zones and regions with lowest uptake observed in Southern (4.73%), Central (5.99%), Western (7.22%) and Zanzibar (8.76%) zones of Tanzania. Specifically, from these regions Geita (2.80%), Mtwara (2.90%), Singida (3.61%) and Kaskazini Pemba (4.26%). In the TDHS reported that, about distribution of wealth is unevenly distributed across zones/regions which can explain the reasons for lowest uptake in some of the zones/regions [35]. Furthermore, cultural and religion aspects differ too with some settings being urbanized which may influence change in cultural norms and values. Notable regional variations in cervical cancer screening uptake were observed, particularly between neighboring regions such as Iringa and Singida. Although both are largely rural, these differences may reflect variations in access to services, program implementation, and community awareness. The higher uptake in regions like Iringa may indicate stronger health system performance or more effective outreach strategies; however, the available data do not allow detailed exploration of these factors.

In this study, we also determined associated predictors on uptake of cervical cancer screening at individual and household levels. The older age, education levels, marital status, access to source of information such as internet and radio, employed for cash, and being covered by health insurance were individual level’s predictors that significantly associated with uptake of cervical cancer screening. Women aged 40 years and above, those with at least primary education level, access to internet and radio in the past 12 months, reported to be employed for cash, use health insurance to access health services were more likely to report utilizing cervical cancer screening services than their counterparts. These findings are similar with other studies elsewhere [21, 22, 25–27, 29, 30]. The study by Ng’ang’a et al. and Isabirye et al. reported that older women are more likely to utilize cervical cancer screening services than younger women [25, 27]. This could be due to general health literacy on age being a risk factor to most of chronic conditions as well as non-communicable diseases. As it was reported in the study in Kenya by Gatume et al., a higher proportion of older women had better knowledge on risk factors for cervical cancer than younger women [36]. In addition, generational differences in access to health information and communication channels may play a role, as younger women may be less reached by traditional health messaging. These findings suggest the need for age-specific strategies, including leveraging peer influence and digital media platforms, to improve screening uptake among younger women.

Education is one of the key social determinants of health with those with higher education level displaying cancer literacy and their uptake of cervical cancer screening services [25, 27, 36, 37]. In addition, women empowered on areas of education, decision making, and access to finance may facilitate their health seeking behavior including uptake to preventive health services such as cervical cancer screening [25, 27, 36, 37]. Furthermore, availability of relevant and appropriate health information reported to positively influence community/women utilizing health care services [38]. Studies document that availability of health information through radio, television, and internet associated with high uptake of preventive services such as cervical cancer screening which is similar to what we found in this study [26, 38]. However, lack of health information specifically on cervical cancer screening service remains a barrier in low uptake of this service in Tanzania [29].

In this study, we observed inequality on uptake of cervical cancer screening services that can be explained by wealth quintile and urbanization level through spatial analysis. Women who participated from regions/zones with relatively poor/middle wealth quintiles and those from rural communities were less likely to uptake cervical cancer screening services. High income and urbanization have been linked to accessibility, affordability, and availability of health services in which suggests women from high income households and those from urban settings are more likely to initiate or demand for preventive health services such as cervical cancer screening compared to their counterparts. The findings are similar to other studies elsewhere [25–27, 30]. To the best of our knowledge, this is the first study in Tanzania that looked at spatial distribution at the country level on the uptake of cervical cancer screening and multi-level predictors influencing it.

Being a secondary data analysis, we could not explore availability of cervical cancer screening which may vary across the regions/zones. However, cervical cancer screening is provided free assuming all eligible women have equal access to services. So, the current findings highlight the areas which need close attention and structured interventions which are culturally sound and acceptable.

### Conclusions and future implications

Generally, the uptake of cervical cancer screening is low among women aged 30 to 49 years with significant variation across regions and zones. The significant predictors at individual/household levels which associate with uptake of cervical cancer screening are older age, high education level, having health insurance, having source of income (employed for cash), and access to source of information such as radio, television, and internet. Women from urban and with high income status were more likely to utilize cervical cancer screening services than their counterparts. Furthermore, integrating cervical cancer screening with existing health programs, such as HPV vaccination initiatives, may provide an opportunity to improve coverage through more coordinated service delivery.Achieving the WHO target of screening 70% of eligible women by 2030 will require context-specific, multi-level interventions that address both structural and behavioral bar.

## Data Availability

The datasets generated during the current study are available in the Demographic and Health Survey Program repository: http://dhsprogram.com/data/available-datasets.cfm and are accessible after registration on the website. The authors did not have special access privileges.
